# An Immunosuppressant Peptide from the Hard Tick *Amblyomma variegatum*

**DOI:** 10.3390/toxins8050133

**Published:** 2016-05-03

**Authors:** Yufeng Tian, Wenlin Chen, Guoxiang Mo, Ran Chen, Mingqian Fang, Gabriel Yedid, Xiuwen Yan

**Affiliations:** 1 Clinical Laboratory, People’s Hospital of Rizhao, 126th Taian Road, Rizhao 276826, Shandong, China; tyf6909@163.com; 2 College of Life Sciences, Nanjing Agricultural University, Weigang #1, Nanjing 210095, Jiangsu, China; mgx@njau.edu.cn (G.M.); frank.sledge@hotmail.com (R.C.); 2015116044@njau.edu.cn (M.F.); gyedid03@yahoo.com (G.Y.); 3 Yunnan Clinical Research Center of Breast Cancer, The Third Affiliated Hospital of Kunming Medical College, Kunming 650032, China; chenwenlin@aliyun.com

**Keywords:** hard tick, blood sucking, salivary glands, immunosuppressant peptide, *Amblyomma variegatum*

## Abstract

Ixodid ticks are well known for spreading transmitted tick-borne pathogens while being attached to their hosts for almost 1–2 weeks to obtain blood meals. Thus, they must secrete many immunosuppressant factors to combat the hosts’ immune system. In the present work, we investigated an immunosuppressant peptide of the hard tick *Amblyomma variegatum*. This peptide, named amregulin, is composed of 40 residues with an amino acid sequence of HLHMHGNGATQVFKPRLVLKCPNAAQLIQPGKLQRQLLLQ. A cDNA of the precursor peptide was obtained from the National Center for Biotechnology Information (NCBI, Bethesda, MD, USA). In rat splenocytes, amregulin exerts significant anti-inflammatory effects by inhibiting the secretion of inflammatory factors *in vitro*, such as tumor necrosis factor-alpha (TNF-α), interleukin-1 (IL-1), interleukin-8 (IL-8) and interferon-gamma (IFN-γ). In rat splenocytes, treated with amregulin, compared to lipopolysaccharide (LPS) alone, the inhibition of the above inflammatory factors was significant at all tested concentrations (2, 4 and 8 µg/mL). Amregulin shows strong free radical scavenging and antioxidant activities (5, 10 and 20 µg/mL) *in vitro*. Amregulin also significantly inhibits adjuvant-induced paw inflammation in mouse models *in vivo.* This peptide may facilitate the ticks’ successful blood feeding and may lead to host immunotolerance of the tick. These findings have important implications for the understanding of tick-host interactions and the co-evolution between ticks and the viruses that they bear.

## 1. Introduction

Ixodid ticks are important arthropod vectors that transmit pathogens to other animals. The saliva of these ticks contains a cocktail of dozens of different bioactive molecules, including coagulation inhibitors, immunomodulatory molecules and, in many ixodid genera, toxins, which may cause acute, progressive and potentially fatal muscle paralysis [1]. It has recently been suggested that when accounting for the composition of tick saliva, the venomous interactions that ticks have with their hosts and the distinguishable differences between human (non-venomous) and tick salivary proteins, ticks should be considered venomous ectoparasites [2]. Some cattle and sheep can develop immunity to tick-induced paralysis [3,4]. In humans, such paralysis occurs mainly in people with a low or reduced immune response (usually children and the elderly) [5,6,7]. Thus, we speculate that immunomodulatory molecules may also play a role when these toxins affect their hosts.

Medically-significant tick-borne diseases include Lyme disease [8,9], babesiosis [10,11], granulocytic ehrlichiosis [12,13,14] and encephalitis [15]. The severe disease Crimean—Congo hemorrhagic fever, for which outbreaks have been documented throughout much of Africa, Asia and Europe, has an approximately 30% fatality rate [12,13,16]. The ixodid *A. variegatum* has been identified as a vector for pathogens, such as *Rickettsia* spp. (human rickettsial infections) [17] and *Ehrlichia ruminantium* (heart water disease) [18]. Novel salivary protein families of *A. variegatum* have also recently been identified. Because most of these proteins have no known function, functional analysis of these proteins with the aim of discovering novel pharmacologically-active compounds is of considerable interest.

Several studies have reported that secretions (mainly proteins) extracted from tick saliva and salivary glands may inhibit host humoral immunity, as well as B- and T-cell responses to tick-transmitted pathogens [19,20,21]. Moreover, these proteins may downregulate host immunity [22,23], alter host blood flow [24,25] or even inhibit host inflammatory responses [26,27,28]. Tick saliva-enhanced transmission has also been demonstrated for several viral and bacterial pathogens, including the tick-borne encephalitis virus and *Borrelia burgdorferi* spp*.*, the causative agent of Lyme disease [21,29,30]. It has been suggested that the immunomodulatory mechanisms of different proteins found in tick salivary gland secretions may share many similarities, despite the presence of marked molecular polymorphisms. Previous research has shown that tick saliva and salivary gland extracts, from which several cytokine-binding peptides and proteins have been identified, can inhibit host inflammatory responses by modulating the host’s cytokine secretions or directly downregulating cytokine activity via extract/cytokine interaction [19,20,31,32]. In this study, a cDNA (Accession BK007793.1) encoding the precursor of a peptide identified from the salivary glands of the hard tick *A. variegatum* and named amregulin was obtained from NCBI. This peptide suppresses the host inflammatory response by inhibiting cytokine secretion and detoxifying reactive oxygen species. Furthermore, the reliability of amregulin's immunosuppression function *in vivo* was also examined in this work.

## 2. Results

### 2.1. Sequence Analysis and the Effects of Amregulin on Cytokine Secretions Induced by LPS

**Figure 1:**
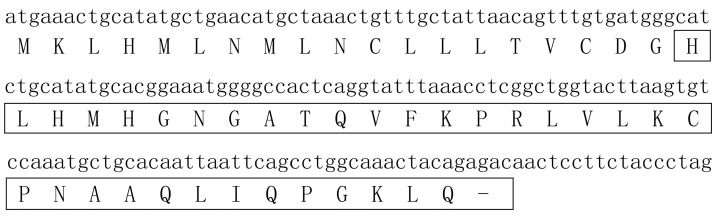
Nucleotide sequence encoding amregulin and the deduced amino acid sequence of the precursor peptide. The region corresponding to mature amregulin is boxed. The bar (–) indicates a stop codon.

The cDNA of amregulin encodes a precursor protein composed of 59 amino acid (aa) residues with the sequence of MKLHMLNMLNCLLLTVCDGHLHMHGNGATQVFKPRLVLKCPNAAQLIQPGKLQRQLLLQ. The mature peptide is composed of 40 residues with the sequence of HLHMHGNGATQVFKPRLVLKCPNAAQLIQPGKLQRQLLLQ (Figure 1).

**Figure 2:**
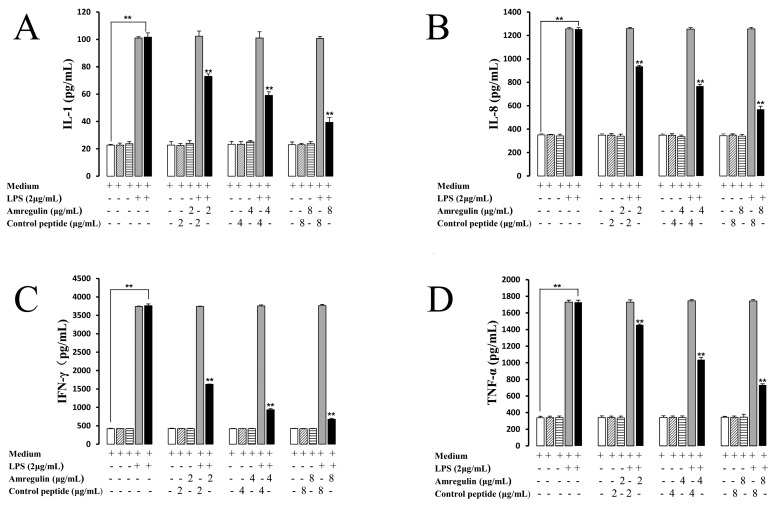
Amregulin significantly inhibits the cytokine secretions induced by lipopolysaccharide (LPS) in rat splenocytes: IL-1 (**A**); IL-8 (**B**); IFN-γ (**C**); and TNF-α (**D**); ** *p* < 0.01 (*n* = 3).

To evaluate the immunosuppression capabilities of amregulin, its effects on IL-1, IL-8, IFN-γ and TNF-α secretions induced by LPS in rat splenocytes were tested. Four different concentrations (0, 2, 4 and 8 µg/mL) of amregulin were used to stimulate rat splenocytes, either alone or in concert with LPS. Treatment with LPS alone strongly induced the secretion of IL-1, IL-8, IFN-γ and TNF-α, whereas amregulin markedly inhibited these LPS-induced cytokine secretions in a dose-dependent manner (Figure 2A–D). The inhibitory effects of amregulin on LPS-induced cytokine secretions, especially IFN-γ, became more evident with increasing concentrations. At a dose of 8 µg/mL of amregulin, the LPS-induced secretion of IFN-γ was decreased nearly five-fold compared to that of the control (699 ± 8.5 pg/mL *vs.* 3720 ± 25.3 pg/mL). TNF-α, IL-1 and IL-8 were also significantly inhibited by amregulin, as compared to the LPS-treated control groups. A scrambled control peptide was also used as a control. At every test concentration, the control showed no effects on the secreted amounts of the different cytokines. These results collectively indicate that amregulin markedly inhibits the LPS-induced secretions of four different inflammatory cytokines in rat splenocytes in a concentration-dependent manner.

### 2.2. Antioxidant Activities of Amregulin

#### 2.2.1. Free Radical Scavenging Activity

Assays with 2,2-diphenyl-1-picrylhydrazyl (DPPH) and 2,2′-azinobis 3-ethylbenzothiazoline-6-sulfonic acid (ABTS^+^) radicals are commonly used to evaluate the antioxidant capacity of biomolecules due to their maneuverability, relative stability and good reproducibility [33]. Reactions with antioxidant molecules convert these colored free radicals into colorless products. Generally, these two free radical systems are correlated with each other, with similar chemical properties of hydrogen/electron donation and tissue damage. Here, we quantified the amounts of reduced DPPH and ABTS^+^ by measuring decreases in absorbance at 517 and 734 nm, respectively. Amregulin showed a strong, concentration-dependent scavenging ability toward ABTS^+^, as compared to both a negative (scrambled control peptide) control and a positive butylated hydroxytoluene (BHT) control, but not toward DPPH, as compared to a BHT positive control. The scrambled control peptide showed very limited free radical scavenging activity (even at a concentration of 20 µg/mL). Nevertheless, amregulin’s ability to scavenge DPPH free radicals also became stronger with increasing concentrations. At concentrations of 10 and 20 µg/mL, amregulin also showed a strong, concentration-dependent ability to quench Nitric Monoxide (NO), which corresponds to its ability to reduce DPPH and ABTS^+^ free radicals. These results indicate that amregulin possesses strong free radical scavenging capacity (Table 1).

#### 2.2.2. Fe^3+^ Reducing Power

We determined the capacity of amregulin to reduce ferric ions (Fe^3+^) by a Fe^3+^–Fe^2+^ transformation method. As shown in Table 1, amregulin showed significant, concentration-dependent reducing power compared to the negative control (scrambled control peptide) and positive standard (BHT). After the 5, 10 or 20 µg/mL of amregulin treatments, the absorbance at 700 nm was decreased by 0.13 ± 0.02, 0.10 ± 0.03 and 0.08 ± 0.01, respectively, compared to the positive control, BHT (0.27 ± 0.06). All of these results collectively indicate that amregulin possesses strong antioxidant activity, even stronger than that of BHT at the minimum test concentration (Table 1), and that amregulin neutralizes free radicals by serving as an electron donor to form stable products with reactive species.

### 2.3. Inhibition of Adjuvant-Induced Paw Inflammation by Amregulin

**Figure 3:**
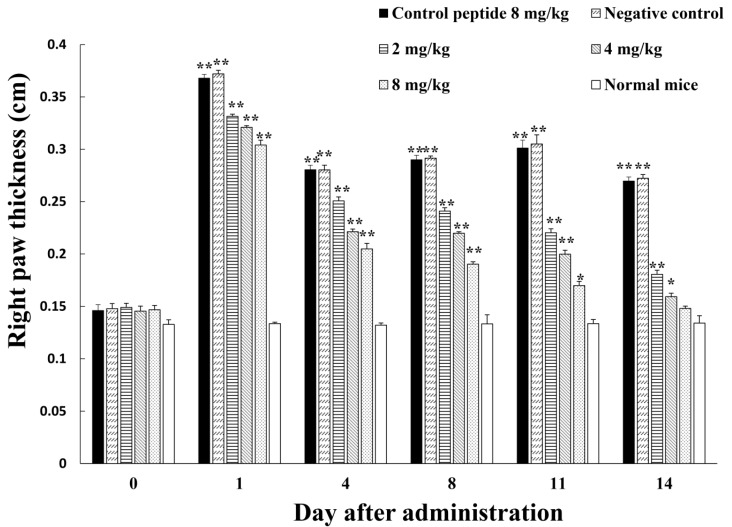
Inhibition of adjuvant-induced mouse paw inflammation by amregulin. All values are means ± SD (*n* = 12). The values for the paw thickness treated by amregulin are significantly different from the values for the controls; * *p* < 0.05, ** *p* < 0.01, respectively.

In light of the *in vitro* inhibitory effects of amregulin on host secretion of diverse inflammatory factors, as well as its free radical scavenging capabilities, we used an induced inflammation model (Freund’s complete adjuvant) to evaluate the potential anti-inflammatory ability of amregulin *in vivo*. As indicated in the Experimental Section, the basal footpad thickness of different male Kunming mice after treatment was selected as an indicator of inflammation. Along with the increments in treatment time, the anti-inflammatory effects of amregulin were enhanced with increasing concentrations, whereas the scrambled control peptide (8 mg/kg bodyweight) showed nearly no effects on inflammation. Significant anti-inflammatory effects were present in all mice treated with different concentrations (2, 4 or 8 mg/kg body weight) of amregulin; the paw inflammation was significantly decreased compared to that of the negative controls. The mice treated with 4 and 8 mg/kg body weight recovered to normal status after 14 days of administration. These results collectively indicate that amregulin inhibits hind paw inflammation in mice in a concentration-dependent manner (Figure 3).

## 3. Discussion

### 3.1. Effects of Amregulin on Cytokine Secretion Induced by LPS

It has been widely recognized that investigations of the interactions between blood-feeding arthropods and their hosts can uncover transmission mechanisms for vector-borne pathogens [34]. As blood-feeding arthropods, ticks may be an excellent animal model for host/parasite relationship studies. Hard ticks ingest blood as well as lymph and lysed tissues, whereas soft ticks feed only on blood. Hard ticks take a very long time (many days) to complete their blood-feeding meals. However, soft ticks need hours or less. Because their feedings last 1–2 weeks, hard ticks were selected as an extreme example to conduct research. To successfully obtain blood meals (and ultimately survive), hard ticks secrete immunosuppressant substances to defeat host immune defenses. Previous research has shown that tick saliva or salivary gland secretions have the potential to inhibit host immune responses and to enhance the transmission of tick-borne pathogens. More than 10 proteins from tick salivary glands have been found to exert immunomodulatory functions. One of them, Salp15, is a major immunomodulatory protein in *Ixodes scapularis* saliva. Salp15 inhibits T-cell receptor ligation-induced T-cell signaling by binding to CD4 [35,36]. Compared to Salp15, amregulin is a small peptide, which makes it a promising candidate for a medicine template. Another two immunoregulatory peptides (hyalomin-A and -B) identified from the salivary glands of *Hyalomma asiaticum* also show similar host inflammatory response suppression by modulating cytokine secretion and detoxifying reactive oxygen species [37]. Amregulin shares some similar characteristics with hyalomin-A and -B, but has different effects on LPS-induced cytokine secretion. Whereas hyalomin-A and -B strongly inhibit the secretion of IFN-γ, monocyte chemotactic protein-1 (MCP-1) and TNF-α, amregulin affects IL-1 and IL-8 in addition to IFN-γ and TNF-α. The different targets indicate the different roles of these salivary peptides.

The progress of tick feeding not only causes direct injury to the host’s skin, but also provides opportunities for disease-causing organisms to be ingested or expelled by the parasite. Thus, the host inflammatory and immune responses must be initiated. IL-1 was first discovered because of its strong pro-inflammatory effects. It induces a complex network of pro-inflammatory cytokines that initiate and regulate inflammatory responses via the expression of various integrins on leukocytes and endothelial cells. IL-8 induces chemotaxis of target cells, primarily neutrophils, as well as other granulocytes, causing them to migrate toward the site of infection. In humans, IL-8 is believed to play an important role in the pathogenesis of bronchiolitis [38], which is caused by viral infections. This finding indicates that IL-8 may participate in the passage of tick-borne viruses. Some ixodid species can transmit several (e.g., *I. ricinus*, *A. variegatum*) or many (*I. uriae*) such viruses [39]. Determining the molecular adaptations that allow these viruses to infect and replicate in both vector and host cells, as well as identifying the principal ecological determinants of virus survival are unsolved key challenges. The present work demonstrated that amregulin markedly inhibits LPS-induced secretion of cytokines, such as IL-1, IL-8, IFN-γ and TNF-α, in a concentration-dependent manner. TNF-α is one of the cytokines that makes up the acute phase reaction. Its primary role is regulating diverse immune cells, and increasing its local concentration causes the typical signs of inflammation: heat, swelling, redness and pain. Inhibition of TNF-α by amregulin suppresses the initial host response to these parasites and, thus, is beneficial for ticks taking their blood meals. Furthermore, amregulin also inhibits the only member of the type II class of interferons, IFN-γ, which inhibits viral replication directly and activates macrophages, enhancing their ability to kill intracellular organisms. Mycobacteria inhibit phagolysosome maturation, which may further affect macrophage function. IFN-γ secreted from Th1 helper cells activates macrophages in this pathological process [40]. The effects of amregulin on the dendritic cells were also tested, it showed that no affect both on the maturation and function of amregulin compare to the positive control LPS (Supplementary Materials). The inhibition of different pro-inflammatory cytokines in our study showed that amregulin may help ticks successfully obtain blood from their hosts.

### 3.2. Antioxidant Activities of Amregulin

Beyond its strong antioxidant activity, amregulin was found to scavenge free radicals in a concentration-dependent manner. It has been demonstrated that ABTS radicals react with phenols to form purple products with a broad absorbance of approximately 550 nm [41,42]. Of the essential amino acids, only tyrosine contains a phenol side chain, and mature amregulin contains only a single tyrosine residue. However, the strong free radical-scavenging ability and antioxidant activity of amregulin would be beneficial for the protection of tick-borne pathogens from host oxidant immune stress, as there is much evidence of overlap between the production of oxidants and the inflammatory response. During their blood meals, ticks have a difficult time coping with the toxic nature of the host inflammatory response, which involves several different oxidative molecules, including iron. Although necessary for a number of physiological processes, high amounts of iron are potentially toxic because iron can also participate in the formation of free radicals, which may cause cellular damage or even death. Tick feeding causes direct damage to the skin of the host, the release of iron and the formation of free radicals, all of which contribute to the host’s inflammatory response. Our results demonstrated that amregulin shows a strong ability to overcome potential damage from Fe^3+^ iron, indicating that amregulin may be an important peptide in the blood feeding process of *A. variegatum.*

### 3.3. Inhibition of Adjuvant-Induced Paw Inflammation by Amregulin

Beyond showing the antioxidant and anti-inflammatory effects of amregulin at molecular and cellular levels, we also performed *in vivo* research by using an adjuvant-induced inflamed mouse paw model to demonstrate the potential of amregulin to suppress inflammation and noted concentration-dependent inhibition of inflammation. At Day 11, the group treated with 8 mg/kg amregulin differed significantly from the negative control group. At Day 14, the same group showed no difference in paw thickness from normal mice. The 4 mg/kg amregulin-treated group showed greatly decreased paw inflammation, whereas the scrambled control peptide showed virtually no effects on inflammation. Each amregulin-treated group showed that the administration of this peptide was able to substantially control the development of inflammation. This result demonstrates the biological significance of this immunosuppressant peptide derived from hard tick salivary glands and contributes to further understanding of the mechanism underlying hard tick immunosuppressive properties against host inflammatory responses.

Although the synthetic peptide may not be the same as the endogenous form because of posttranslational modifications, such as glycosylation or interactions with other proteins, or may even form a dimer via the cys residue, our findings may still provide some implications for understanding the mechanisms of successful tick hematophagy and tick-host interactions.

## 4. Conclusions

The present work focused on the peptide amregulin, which exerts significant anti-inflammatory effects by inhibiting host secretion of the inflammatory factors TNF-α, IL-1, IL-8 and IFN-γ. It also shows strong antioxidant properties. Amregulin effectively inhibited the adjuvant-induced inflammation of mouse paws *in vivo*. Together, the results of this study suggest that the biological activities of amregulin facilitate successful parasite feeding.

## 5. Experimental Section

### 5.1. cDNA, Peptide and Cytokine Assays

A cDNA (Accession BK007793.1) encoding the precursor of amregulin was obtained from the National Center for Biotechnology Information (NCBI, Bethesda, MD, USA) [19]. The cDNA was cloned from the salivary glands of the hard tick *A. variegatum*. The protein precursor encoded by the cDNA was analyzed by SignalP 4.0 (The Center for Biological Sequence Analysis, Technical University of Denmark, Copenhagen, Denmark, 2016 [43]) to predict signal and mature peptides. Amregulin (HLHMHGNGATQVFKPRLVLKCPNAAQLIQPGKLQRQLLLQ) and a scrambled control peptide (VLVVAGNGATQVAAPRLVLKLPNAAQLIQIGELQRQLALG) were synthesized by GL Biochem Ltd. (Shanghai, China). Analysis with high performance liquid chromatography (HPLC) and mass spectrometry confirmed that its purity was higher than 98%. The peptide was dissolved in phosphate-buffered saline (PBS; 0.1 M, pH 6.0) solution at a concentration of 2 mg/mL and stored at −20 °C. This stock solution was used to prepare samples for the cytokine assays.

The wells of a 96-well plate were seeded with a suspension of splenocytes from Wistar rats in RPMI 1640 medium (Gibco Life Technologies, Grand island, NY, USA) supplemented with 5% fetal bovine serum and 100 U/mL penicillin and streptomycin. A total of 6 × 10^6^ cells (100 µL per well) were incubated at 37 °C and 3.5% CO_2_. Different concentrations of the peptide were prepared by taking 20-µL samples of previously prepared peptide stock solution and dissolving them in different volumes of RPMI 1640 medium with LPS (final concentration, 2 µg/mL, Sigma, Saint louis, MO, USA). These different peptide solutions were then co-cultured with splenocytes. After a 48-h cultivation, culture supernatants were harvested and stored at −70 °C. All combinations were replicated three times. Cytokine assays for IL-1, IFN-γ, IL-8 and TNF-α were conducted by using antibody-sandwich ELISAs (Adlitteram Diagnostic Laboratories, Inc., Beijing, China) according to the manufacturer’s instructions.

### 5.2. Free Radical Scavenging Activity

#### 5.2.1. DPPH Scavenging

We determined free radical scavenging activity by using a stable DPPH radical (Sigma), slightly modifying a previously-reported method [41,42]. The assay mixture contained 1.9 mL of 5 × 10^5^ M DPPH radical dissolved in ethanol and 0.1 mL of sample solution (concentration range, 0.5–10 mg/mL). Following 30 min of incubation at room temperature, absorbance was red against a blank at 517 nm. The percentage inhibition of DPPH free radicals by the peptide (*I*%) was calculated according to Equation (1):

*I*% = (*A*_blank_ − *A*_sample_) × 100/*A*_blank_ (1)

where *A*_blank_ is the absorbance of the control reaction (reaction mixture minus the test compound) and *A*_sample_ is the absorbance of the test compound. Butylated hydroxytoluene (BHT; Sigma) and a scrambled control peptide were used as a positive and a negative control, respectively.

#### 5.2.2. ABTS^+^ Scavenging

We further determined free radical scavenging activity by measuring the reduction of ABTS^+^ with a GMS10114.4 kit (Genmed Scientifics Inc., Shanghai, China) according to the manufacturer’s instructions, using BHT as a positive control and the scrambled control peptide as a negative control. We used a linear regression analysis to determine the total formation of products (*i.e.*, the reduced form of ABTS and the purple antioxidin-RP1 modification) and the total consumption of the ABTS radical. The concentrations of ABTS^+^ and ABTS were calculated to be ε340 = 4.8 × 10^4^ M^−1^·cm^−1^ and ε415 = 3.6 × 10^4^ M^−1^·cm^−1^, respectively. The purple end product of ABTS^+^ with amregulin was monitored by measuring absorbance at 550 nm.

#### 5.2.3. NO Scavenging

We determined the NO scavenging capacity of amregulin by incubating 5 mM sodium nitroprusside dihydrate (SNP; Sigma) in phosphate-buffered saline (PBS; 0.1 M, pH 6.0) with different concentrations of peptide samples at 25 °C, and 0.5 mL of the incubation solution was withdrawn and mixed with 0.5 mL of Griess reagent (Promega Corporation, Madison, WI, USA) after 20 min. Absorbance was measured at 550 nm. The amount of nitrite was calculated from a standard curve constructed using sodium nitrite.

#### 5.2.4. Fe^3+^ Reducing Power

The reducing power of amregulin against Fe^3+^ was evaluated using the method developed by Wu *et al.* [38]. Varying amounts of tested samples were suspended in distilled water and mixed with 2.5 mL of 0.2 M phosphate buffer (pH 6.6) and 2.5 mL of 1% K_3_Fe(CN)_6_. The mixture was incubated at 50 °C for 20 min, after which 2.5 mL of 10% trichloroacetic acid (TCA) was added to the mixture, and this was followed by centrifugation at 3000 rpm for 10 min. Of the solution, 2.5 mL of the upper layer was mixed with an equal volume of distilled water and 0.5 mL of FeCl_3_ (0.1%), and the absorbance was measured at 700 nm. An increase in the absorbance of the reaction mixture indicated reduction of Fe^3+^. BHT was again used as a positive control.

### 5.3. Adjuvant-Induced Paw Inflammation in Mice

Twenty microliters of vehicle (saline) or Freund’s complete adjuvant (Sigma) was administered to the plantar surface of the right hind paws of male Kunming mice (12/group). The basal footpad thickness of each mouse was measured with a vernier caliper at the beginning of the experiment. Amregulin (2, 4 or 8 mg/kg of mouse body weight) was administered to the rear femoral muscle on alternating days from Day 2 after injection of Freund's complete adjuvant (or saline as a control) to Day 14. The control groups of mice received the same volume of saline (vehicle). Right hind paw thickness was used as an indicator of inflammation and measured with a vernier caliper on Days 0, 1, 4, 8, 11, and 14. The experimental protocols were approved by the Animal Care and Use Committee at Nanjing Agricultural University (identification code: M.110/305; date of approval: 22 April 2015).

### 5.4. Statistical Analysis

All data are presented as means ± SD and were analyzed by a one-way or two-way analysis of variance (ANOVA), followed by Duncan’s test or an unpaired *t-*test. *p*-values less than 0.05 were considered to be statistically significant, whereas *p*-values less than 0.01 were considered extremely statistically significant.

### 5.5. Ethics Statement

The Animal Care and Use Committee of Nanjing Agricultural University evaluated and approved all procedures used in this study. All *in vivo* experiments were performed according to sound practices of laboratory animal management.

## Figures and Tables

**Table 1:** Antioxidant activities of amregulin. ABTS^+^, 2,2′-azinobis 3-ethylbenzothiazoline-6-sulfonic acid; DPPH, 2,2-diphenyl-1-picrylhydrazyl; NO, Nitric Monoxide; NA, Not Available.

Samples	Free Radical Scavenging Capacity (%)			Fe^3+^ Reducing Power (Absorbance at 700 nm)
	ABTS^+^	DPPH	NO	
H_2_O	0 ± 0.0	0 ± 5.4	NA	NA
BHT	73.6 ± 7.9	92.1 ± 10.4	41.7 ± 6.5	0.27 ± 0.06
Amregulin (2.5 µg/mL)	53.1 ± 12.3	39.5 ± 6.9	17.6 ± 4.2	NA
Amregulin (5 µg/mL)	79.9 ± 11.5	63.1± 9.2	30.3 ± 4.9	0.08 ± 0.01
Amregulin (10 µg/mL)	88.3 ± 17.2	70.8 ± 10.4	42.4 ± 6.7	0.10 ± 0.03
Amregulin (20 µg/mL)	93.5 ± 13.6	79.2 ± 13.4	48.7 ± 7.3	0.13 ± 0.02
Control peptide (20 µg/mL)	12.7 ± 3.1	7.6 ± 1.5	3.3 ± 0.8	NA

Values are the means ± SD (*n* = 3). Scrambled control peptide, butylated hydroxytoluene (BHT) (Sigma, Saint louis, MO, USA) and H_2_O were used as controls; equation: *I*% = (*A*_blank_ − *A*_sample_) × 100/*A*_blank_.

## Supplementary Materials

The following are available online at www.mdpi.com/2072-6651/8/5/133/s1, Figure S1: Effect of amregulin on dendritic cells; ***** *p* < 0.05 (*n* = 3).
