# Supplementary material for: An Immunosuppressant Peptide from the Hard Tick Amblyomma variegatum
Source: Toxins (Basel). 2016 May 3;8(5):133. doi: 10.3390/toxins8050133 (PMC4885048; doi:10.3390/toxins8050133)
Supplement: Supplementary file 1 [file toxins-08-00133-s001.pdf]

Supplementary Materials: An Immunosuppressant Peptide from the Hard Tick *Amblyomma variegatum*

Yufeng Tian, Wenlin Chen, Guoxiang Mo, Ran Chen, Mingqian Fang, Gabriel Yedid and Xiuwen Yan

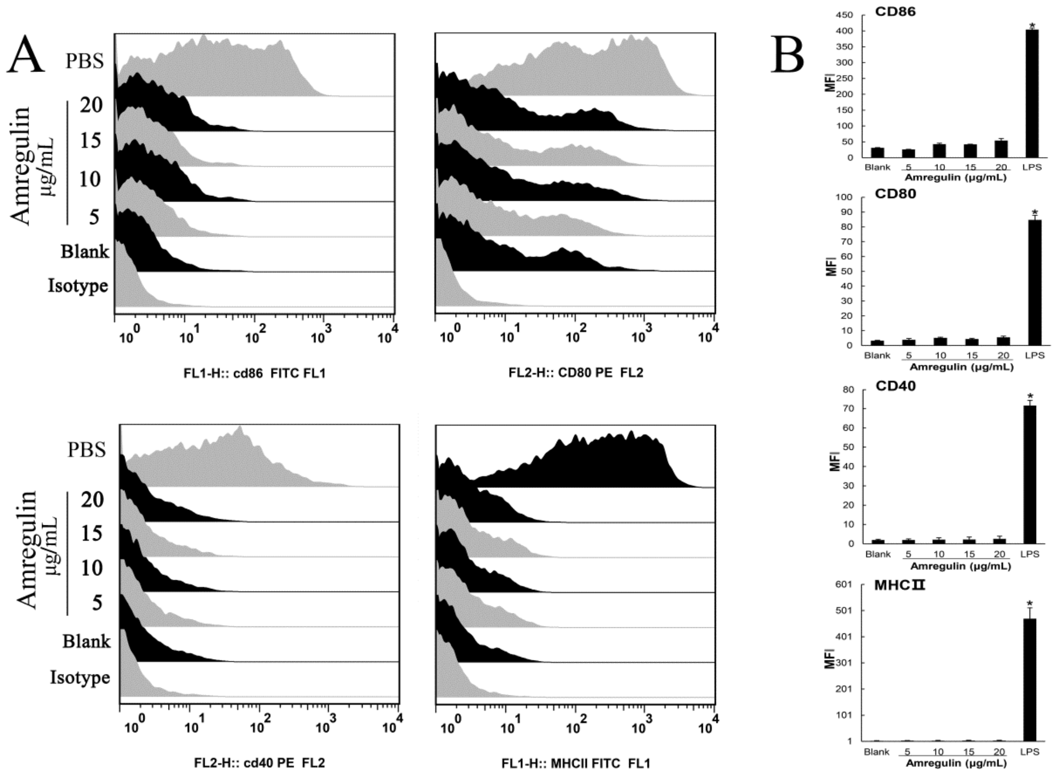

Figure S1. Effect of amregulin on dendritic cells; * $p < 0.05$ ($n = 3$).
